# Hypoxia modulates human mast cell adhesion to hyaluronic acid

**DOI:** 10.1007/s12026-021-09228-x

**Published:** 2021-11-17

**Authors:** Joanna Pastwińska, Aurelia Walczak-Drzewiecka, Elżbieta Kozłowska, Enjuro Harunari, Marcin Ratajewski, Jarosław Dastych

**Affiliations:** 1 Laboratory of Cellular Immunology, Institute of Medical Biology, Polish Academy of Sciences, 90-364 Lodz, Poland; 2 Department of Experimental Immunology, Medical University of Lodz, 92-213 Lodz, Poland; 3 Biotechnology Research Center and Department of Biotechnology, Toyama Prefectural University, 5180 Kurokawa, Imizu, Toyama, 939-0398 Japan; 4 Laboratory of Epigenetics, Institute of Medical Biology, Polish Academy of Sciences, 90-364 Lodz, Poland

**Keywords:** Mast cells, Adhesion, Hyaluronic acid, Hypoxia, CD44, HMMR/RHAMM

## Abstract

Hypoxia is an inherent factor in the inflammatory process and is important in the regulation of some immune cell functions, including the expression of mast cell pro- and anti-inflammatory mediators. Hypoxia also influences cell adhesion to the extracellular matrix (ECM). Hyaluronic acid is one of the major components of the ECM that is involved in inflammatory and tissue regeneration processes in which mast cells play a prominent role. This prompted us to investigate the effects of hypoxia on the expression of hyaluronic acid receptors in mast cells and mast cell adhesion to this ECM component. We found that human LAD2 mast cells spontaneously adhered to hyaluronic acid in a CD44-dependent manner and that reduced oxygen concentrations inhibited or even completely abolished this adhesion process. The mechanism of hypoxia downregulation of mast cell adhesion to hyaluronic acid did not involve a decrease in CD44 expression and hyaluronidase-mediated degradation of adhesion substrates but rather conformational changes in the avidity of CD44 to hyaluronic acid. Hypoxia-mediated regulation of mast cell adhesion to extracellular matrix components might be involved in the pathogenic accumulation of mast cells observed in the course of certain diseases including rheumatoid arthritis and cancer.

**Supplementary Information:**

The online version contains supplementary material available at 10.1007/s12026-021-09228-x.

## Introduction

Oxygen is an element that is necessary for the proper function of cells in living organisms. The oxygen concentration in various tissues is significantly lower than that in atmospheric conditions; e.g., in venous blood, it is approximately 5.3%, while in the superficial region of the skin, it is only 1.1%. Under physiological conditions, the prevailing concentration in a specific tissue is referred to as “tissue normoxia” or “physioxia,” while any decrease in oxygen partial pressure below these values is known as “hypoxia” [1]. Certain pathological processes result in local hypoxia. Thus, hypoxia frequently occurs in tissues as a result of ongoing inflammation [2]. The tumor formation process, which can be considered a special type of inflammation, is also associated with local hypoxia [3, 4]. In vitro cell culture incubators use an oxygen concentration corresponding to that in the air, which is also frequently referred to as “normoxia.” Since cell lines have been derived and cultured under such conditions, lowering the oxygen concentration to physioxic levels may be treated by these cells as hypoxia and is frequently applied as an in vitro model of hypoxia [1]. Oxygen-sensing prolyl hydroxylases (PHDs) and hypoxia-inducible factors (HIFs), such as HIF-1α and HIF-2α, are critical for initiating the cellular response to hypoxia, which includes changes in cell metabolism as well as other cellular functions, including remodeling of the extracellular matrix (ECM), cell adhesion, and migration [5–8].

Mast cells (MCs) are considered one of the most important elements regulating the initiation, amplification, and resolution of inflammatory processes [9, 10]. Proper execution of their functions in regulating inflammation also depends on various cell adhesion receptors on their surface, including integrins, selectins, and cadherins [11]. Human MCs also express CD44, a major receptor for hyaluronic acid (HA) [12]. CD44 does not always bind to HA, and this process is state-dependent. CD44 can assume a nonbinding form, a binding form only under the influence of a specific stimulus, and a constitutively binding form [13]. Hypoxia has been reported to upregulate the gene and surface protein expression of CD44 in human dendritic cells [14], as well as in the human gastric cancer cell lines SGC-7901 and BGC-823 [15].

Previously, we observed an increase in the adhesion of MCs to fibronectin under the influence of short-term exposure to hypoxia [8]. In this study, we investigated the influence of hypoxic conditions on the adhesion of the human MC line LAD2 to HA. Our results revealed that hypoxia downregulates MC adhesion to HA.

## Materials and methods

### Cell line and cell culture

The human MC line LAD2 was a gift from Dr. A. S. Kirshenbaum (National Institutes of Health, Bethesda, MD) [16]. Cells were cultured at 37 °C in 5% CO_2_ and passaged weekly to maintain a 500,000 cells/mL density. Dedicated medium consisted of StemPro-34 serum-free medium (Gibco) supplemented with StemPro-34 Nutrient Supplement (Gibco), 2 mM l-glutamine (Sigma-Aldrich), 100 U/mL penicillin/100 μg/mL streptomycin (Gibco), and 100 ng/mL recombinant human SCF (R&D Systems).

### Quantitative real-time PCR analysis

Before RT-qPCR analysis, cells were cultured for 72 h in normoxia (21% O_2_) or hypoxia (1% O_2_). Total RNA was extracted using TRI Reagent (Sigma-Aldrich) according to the manufacturer’s protocol. cDNA was reverse-transcribed from total RNA (5 μg) using the Maxima First Strand cDNA Synthesis Kit for RT-qPCR (Thermo Fisher Scientific) according to the manufacturer’s protocol. Levels of specific gene expression were measured with qPCR on a LightCycler 480 in a 384-well plate using a SYBR Green I Master Mix kit (Roche). RT-qPCR conditions consisted of initial denaturation at 95 °C for 5 min; 45 cycles of 95 °C for 10 s, 55 °C for 10 s, and 72 °C for 20 s; melting curve at 95 °C for 30 s, 72 °C for 45 s, and 97 °C; and cooling at 40 °C for 15 s. Three housekeeping genes (*HPRT1*, *HMBS*, *RPL13A*) served as internal controls. All samples were analyzed in duplicate, and the results are presented as relative gene expression using the ΔΔct method. *HPRT1*, *HMBS*, and *RPL13A* primer sequences were based on the work of Vandesompele et al. [17], while other primers were based on sequences published elsewhere: *CD44s* [18], *RHAMM* [19], *ICAM-1* [20], *LYVE-1* [21], *TLR4* [22], *HYAL1*, *HYAL2* [23], and *HYAL3* [24]. Other sequences were designed using Primer3 software [25] or PrimerBlast [26]. All primer sequences are shown in Table 1.

**Table 1:** Human-specific primer sequences

Gene	Forward primer	Reverse primer	Amplicon
*HPRT*	5ʹ-tgacactggcaaaacaatgca-3ʹ	5ʹ-ggtccttttcaccagcaagct-3ʹ	94
*HMBS*	5ʹ-ggcaatgcggctgcaa-3ʹ	5ʹ-gggtacccacgcgaatcac-3ʹ	64
*RPL13A*	5ʹ-cctggaggagaagaggaaagaga-3ʹ	5ʹ-ttgaggacctctgtgtatttgtcaa-3ʹ	126
*CD44s*	5ʹ-ccaacacctcccagtatga-3ʹ	5ʹ-gcaggtctgtgactgatg-3ʹ	81
*RHAMM*	5ʹ-cagctggaagatgaagaagg-3ʹ	5ʹ-gcatgtagttgtagctgaaaag-3ʹ	137
*ICAM-1*	5ʹ-gacacctttgttagccacc-3ʹ	5ʹ-ccagtgaaatgcaaacagg-3ʹ	143
*LYVE-1*	5ʹ-cctggtgttgcttctcac-3ʹ	5ʹ-gatccccataattctgcatga-3ʹ	106
*TLR2*	5ʹ-ttctcatctcacaaaattgcaaat-3ʹ	5ʹ-ggaaggtaagtccagcaaaatctt-3ʹ	92
*TLR4*	5ʹ-ctgcaatggatcaaggaccag-3ʹ	5ʹ-tgccctgcttatctgaaggtg-3ʹ	82
*HYAL1*	5ʹ-ccaaggaatcatgtcaggc-3ʹ	5ʹ-cccactggtcacgttcag-3ʹ	77
*HYAL2*	5ʹ-ggcttagtgagatggacctc-3ʹ	5ʹ-ccgtgtcaggtaatctttgag-3ʹ	137
*HYAL3*	5ʹ-ctggcatctccatgactacc-3ʹ	5ʹ-cttccatctgtcctggatctc-3ʹ	137
*HYAL4*	5ʹ-gtggacttgctgttatagattgg-3ʹ	5ʹ-gcactttcttcaaaggtcact-3ʹ	169
*HYAL5*	5ʹ-ttgaacactcagcagtctc-3ʹ	5ʹ-aactctgatggcttcccg-3ʹ	72

### Adhesion assay

Before the adhesion assay, cells were cultured for 72 h in normoxia (21% O_2_) or hypoxia (1% or 5% O_2_). Since 1% oxygen yielded more consistent results, it was subsequently used in adhesion, inhibition of adhesion, FACS, and RT-qPCR analyses. LAD2 cells were prepared by three washing cycles and resuspension in a fully supplemented dedicated medium supplemented with 0.1% BSA. Next, 50,000 cells/100 μL/well were seeded and incubated for 1 h in normoxia or hypoxia in separate 96-well plates (Nunc, Thermo Fisher Scientific). Plates were previously prepared for the assay: precoated overnight at 4 °C with 5 mg/mL HA (Merck Millipore), washed three times with HBSS, blocked for 3 h at 37 °C with 5% BSA, and washed again with HBSS before seeding cells. Short-term hypoxia was analyzed by seeding some of the normoxic cells in hypoxia for only 1 h of adhesion. The percentage of adherent cells was determined using the Acid Phosphatase Assay Kit (Abcam, ab83367). We slightly modified the protocol to adapt the assay to 96-well plates instead of using separate tubes. Nonadherent cells were washed away three times with fully supplemented StemPro-34 0.1% BSA, and all remaining steps were performed under normoxia. Adherent MCs were lysed directly in the plate using 80 μL of assay buffer supplemented with 0.1% Triton X-100 per well, which was introduced to improve cell lysis. Reference samples containing 50,000 cells/well were simultaneously prepared in a U-bottom 96-well plate and transferred to wells in test plates after lysis. Next, 50 μL of 5 mM para-nitrophenylphosphate (pNPP) was added to all test and reference samples. Plates were incubated in the dark for 1 h at 25 °C. Finally, 20 μL of stop solution was added to all test and reference samples to stop the reaction, and absorbance was measured at OD 405 nm. Results are presented as the percentage of adherent cells calculated based on reference samples.

### Inhibition of adhesion by antibody blocking of the CD44 receptor

To determine the role of the CD44 receptor in MC adhesion to HA, we used an antibody that was suitable for inhibitory assays (CD44 antibody, Novus Biologicals) and an isotype control (purified rat IgG2a κ isotype control, BD Pharmingen). LAD2 cells were pretreated with 1 μg/mL or 10 μg/mL antibodies for 30 min before the adhesion assay. Concentrations were selected based on the manufacturer’s suggestions and the literature. After preincubation, an adhesion assay was performed as described above.

### Flow cytometry analysis

LAD2 MCs were incubated for 1 h in normoxia (21% O_2_) or hypoxia (1% O_2_) and fixed under the same conditions for 5 min in 500 μL of flow cytometry fixation buffer (R&D Systems). After fixation, MCs were washed in FACS buffer containing 1 × PBS, 1% BSA, and 0.1% sodium azide and were subsequently resuspended in 100 μL of this buffer. Next, MCs were incubated for 30 min with primary nonspecific antibody (mouse IgG1 isotype control, Novus Biologicals) at a 1:1000 dilution, with primary antibody against the RHAMM receptor (RHAMM/CD168 antibody, Novus Biologicals) at a 1:500 dilution followed by incubation in the dark for 30 min at 4 °C with fluorescence-labeled secondary antibody (goat anti-mouse IgG-FITC, Santa Cruz Biotechnology) at a 1:400 dilution, with primary nonspecific antibody (purified rat IgG2a κ isotype control, BD Pharmingen) at a 1:500 dilution, and with primary antibody against the CD44 receptor (CD44 antibody, Novus Biologicals) at a 1:500 dilution, followed by fluorescence-labeled secondary antibody (goat anti-rat Ig-FITC, BD Pharmingen) at a 1:500 dilution. Finally, MCs were washed with FACS buffer and resuspended in 1 mL of FACS buffer. Flow cytometry analysis was performed using a BD LSR Fortessa™ (BD Biosciences).

### Statistical analysis

Statistical procedures were performed with STATISTICA V.13.1 (StatSoft, Tulsa, OK, USA). All data are expressed as the mean ± SEM. The normality of distribution was evaluated using the Shapiro–Wilk test. Student’s *t*-test was used to detect statistically significant differences that are set at *P* < 0.05. In some analyses, Bonferroni correction was incorporated for Student’s *t*-test comparisons.

## Results

### MCs express common hyaluronic acid receptors: CD44, RHAMM, and hyaluronidase HYAL2

First, we analyzed the expression of genes coding for HA receptors and HA-degrading enzymes in LAD2 MCs cultured under standard and hypoxic conditions using RT-qPCR. Investigated genes included the major HA receptor *CD44*, genes coding for alternative HA receptors, such as hyaluronan-mediated motility (*HMMR/RHAMM*), intercellular adhesion molecule 1 (*ICAM-1*), lymphatic vessel endothelial receptor 1 (*LYVE-1*) [27, 28], *TLR2* and *TLR4*, which were reported to interact with low molecular weight HA [27], and five genes encoding *HYAL1-5* hyaluronidases [29]. As shown in Table 2, MCs under atmospheric oxygen concentrations expressed *CD44* and *HMMR/RHAMM* and exhibited very low expression of *ICAM-1*. Interestingly, cells cultured under hypoxia conditions exhibited slightly increased *CD44* expression and significantly decreased expression of *HMMR/RHAMM*. Analysis of expression of the hyaluronidase genes *HYAL1-5* showed that mast cells expressed only *HYAL2* (Table 2), and hypoxic conditions did not significantly change its expression level.

**Table 2:** Relative gene expression of hyaluronic acid receptors and hyaluronidases in LAD2 mast cells under hypoxic conditions. The mRNA expression of genes encoding hyaluronic acid receptors *CD44*, *HMMR/RHAMM*, *ICAM-1*, *LYVE-1*, *TLR2*, and *TLR4* and hyaluronidases *HYAL1*, *HYAL2*, *HYAL3*, *HYAL4*, and *HYAL5* was measured after 72-h incubation in 21% (normoxia) or 1% (hypoxia) oxygen. Mean ± SEM. *P < 0.05, Student’s *t*-test

Gene	Relative gene expression ± SEM	
	Normoxia	Hypoxia
*CD44*	61.13 ± 8.27	86.12 ± 10.60
*HMMR*	34.48 ± 2.60	2.55 ± 0.92*
*ICAM-1*	11.77 ± 0.90	11.72 ± 0.85
*LYVE-1*	0.00 ± 0.00	0.00 ± 0.00
*TLR2*	0.01 ± 0.00	0.02 ± 0.01
*TLR4*	1.52 ± 0.41	1.90 ± 0.13
*HYAL1*	6.24 ± 0.59	7.43 ± 0.25
*HYAL2*	147.84 ± 7.39	120.55 ± 3.93*
*HYAL3*	8.69 ± 1.00	1.85 ± 0.65*
*HYAL4*	0.18 ± 0.06	0.40 ± 0.13
*HYAL5*	0.56 ± 0.11	0.58 ± 0.13

### Hypoxia downregulates MC adhesion to HA

**Fig. 1:**
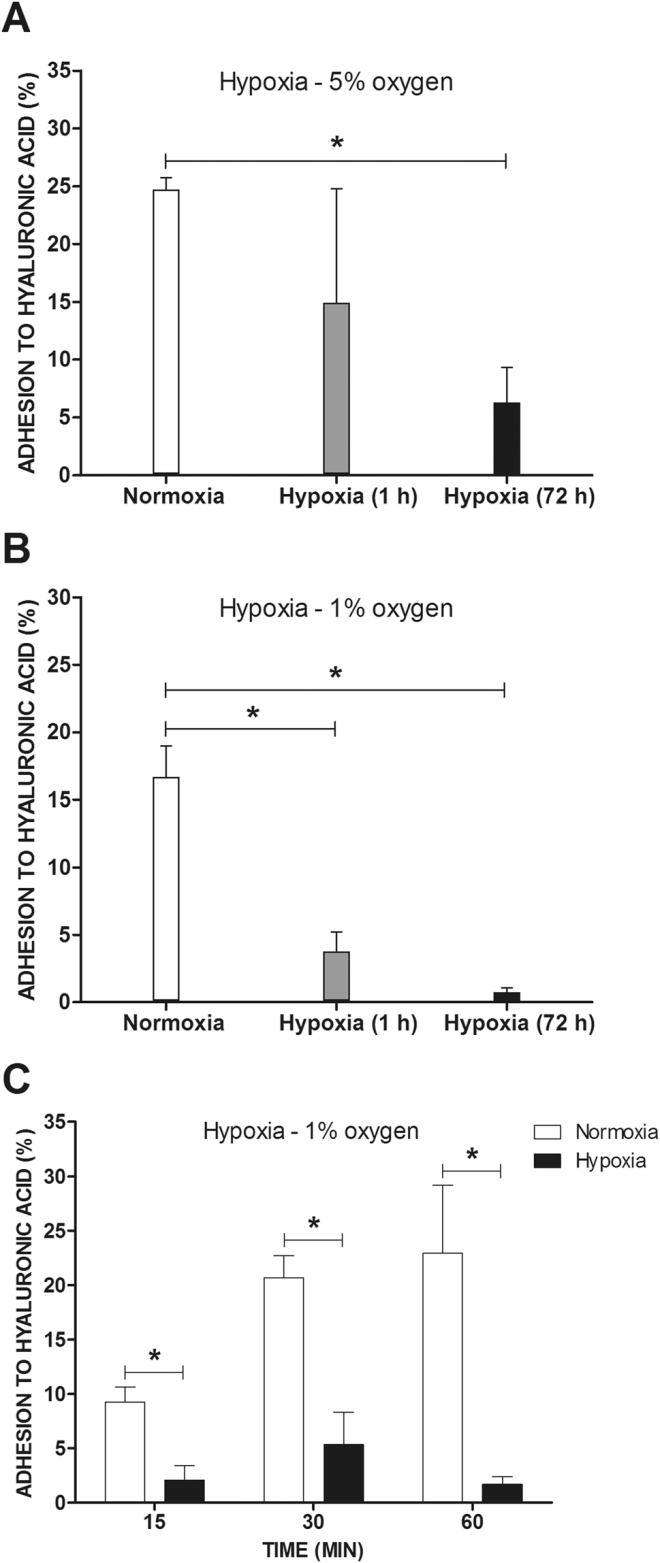
Adhesion of LAD2 mast cells to HA under hypoxic conditions. LAD2 cellular adhesion to HA assessed after 1 h and 72 h of incubation in 21% (normoxia) and 5% (**A**) or 1% (hypoxia) (**B**) oxygen. Kinetics of LAD2 adhesion to HA assessed after 15, 30, and 60 min of incubation in 21% (normoxia) and 1% (hypoxia) oxygen (**C**). Mean ± SEM. *P < 0.05, Student’s *t*-test (**A**, **B**); *P < 0.02, Student’s *t*-test with Bonferroni correction

We examined the adhesion of MCs to HA under normoxic (21% oxygen) and hypoxic (5% or 1% oxygen) conditions. As shown in Fig. 1A–C, MCs spontaneously adhered to HA when cultured under atmospheric oxygen. This adhesion process was observed after 15 min and reached a maximum at 60 min of incubation of MCs in HA-coated plates (Fig. 1C). Under both 5% and 1% oxygen concentration hypoxic conditions, MC adhesion to HA was significantly decreased compared to controls. Hypoxic conditions of 1% oxygen resulted in greater inhibition of MC adhesion to HA than 5% (Fig. 1A, B). Longer (72 h) exposure of MCs to hypoxic conditions prior to their placement in HA-coated plates resulted in greater inhibition (5% oxygen, Fig. 1A) or complete abolishment (1% oxygen, Fig. 1B) of MC adhesion to HA.

### Preincubation of MCs under hypoxic conditions does not affect their subsequent adhesion to HA under normoxic conditions

**Fig. 2:**
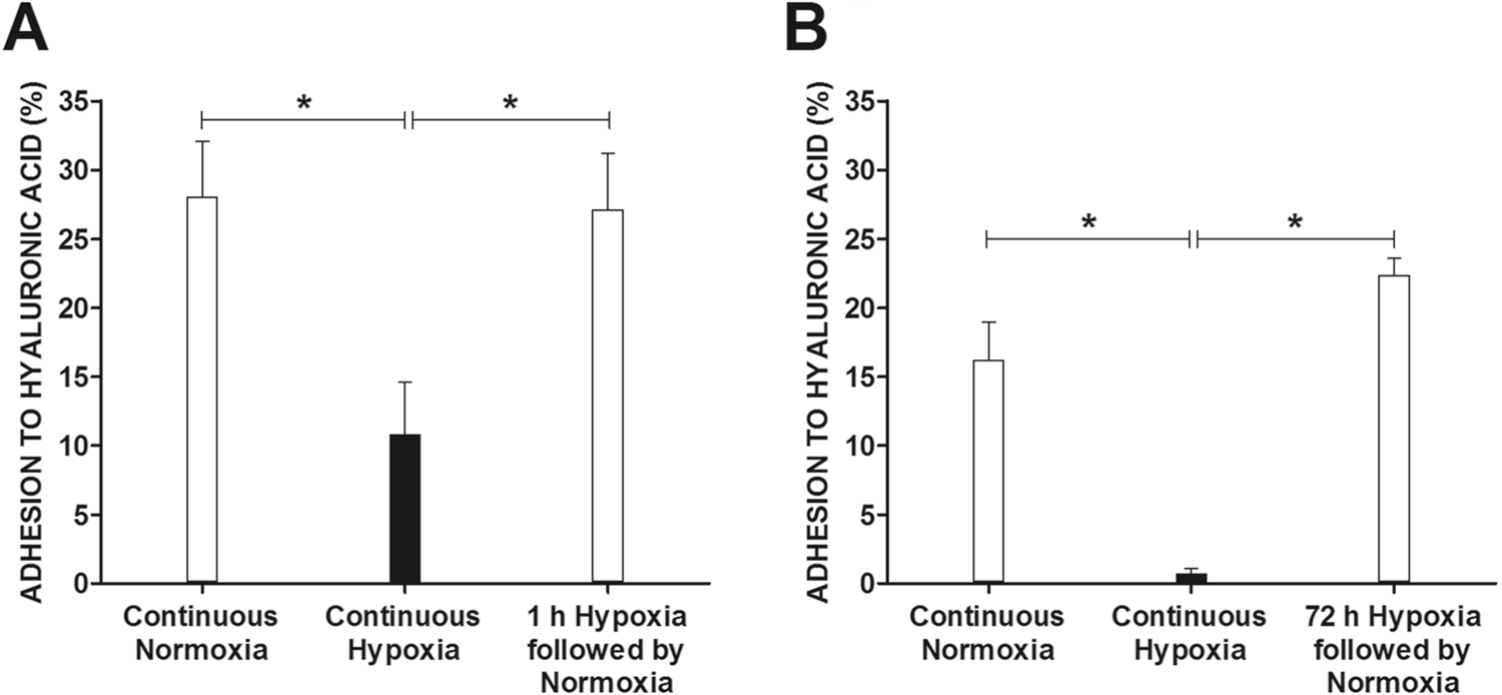
Adhesion of LAD2 mast cells to HA under normoxia after hypoxic conditions. LAD2 mast cell adhesion to HA assessed exclusively in 21% (continuous normoxia) or 1% (continuous hypoxia) oxygen or in 21% oxygen after 1-h (**A**) and 72-h (**B**) incubation in 1% hypoxia (1/72-h hypoxia followed by normoxia). Mean ± SEM. *P < 0.05, Student’s *t*-test

Next, we investigated whether the observed inhibition of MC adhesion to HA by hypoxia was reversible. To this end, MCs were precultured under hypoxic conditions for 1 and 72 h (Fig. 2A and B, respectively) and subsequently placed in HA-coated plates under normoxic conditions. As seen in Fig. 2A, MCs exposed to hypoxic conditions for 1 h before adhesion assay adhered to HA in numbers comparable to unexposed controls in an adhesion assay performed under normoxic conditions but did exhibit significantly lower adhesion when the adhesion assay was also performed under hypoxic conditions (Fig. 2A). MCs preincubated under hypoxic conditions for 72 h completely failed to adhere to HA when the adhesion assay was also performed under hypoxic conditions and adhered in numbers comparable to the control when the adhesion assay was performed under normal atmospheric conditions (Fig. 2B). Thus, the adhesion of MCs to HA is dependent upon atmospheric conditions during the adhesion assay and not on cell culture conditions prior to the adhesion assay.

### CD44 mediates MC adhesion to HA, but hypoxia does not affect CD44 surface expression

**Fig. 3:**
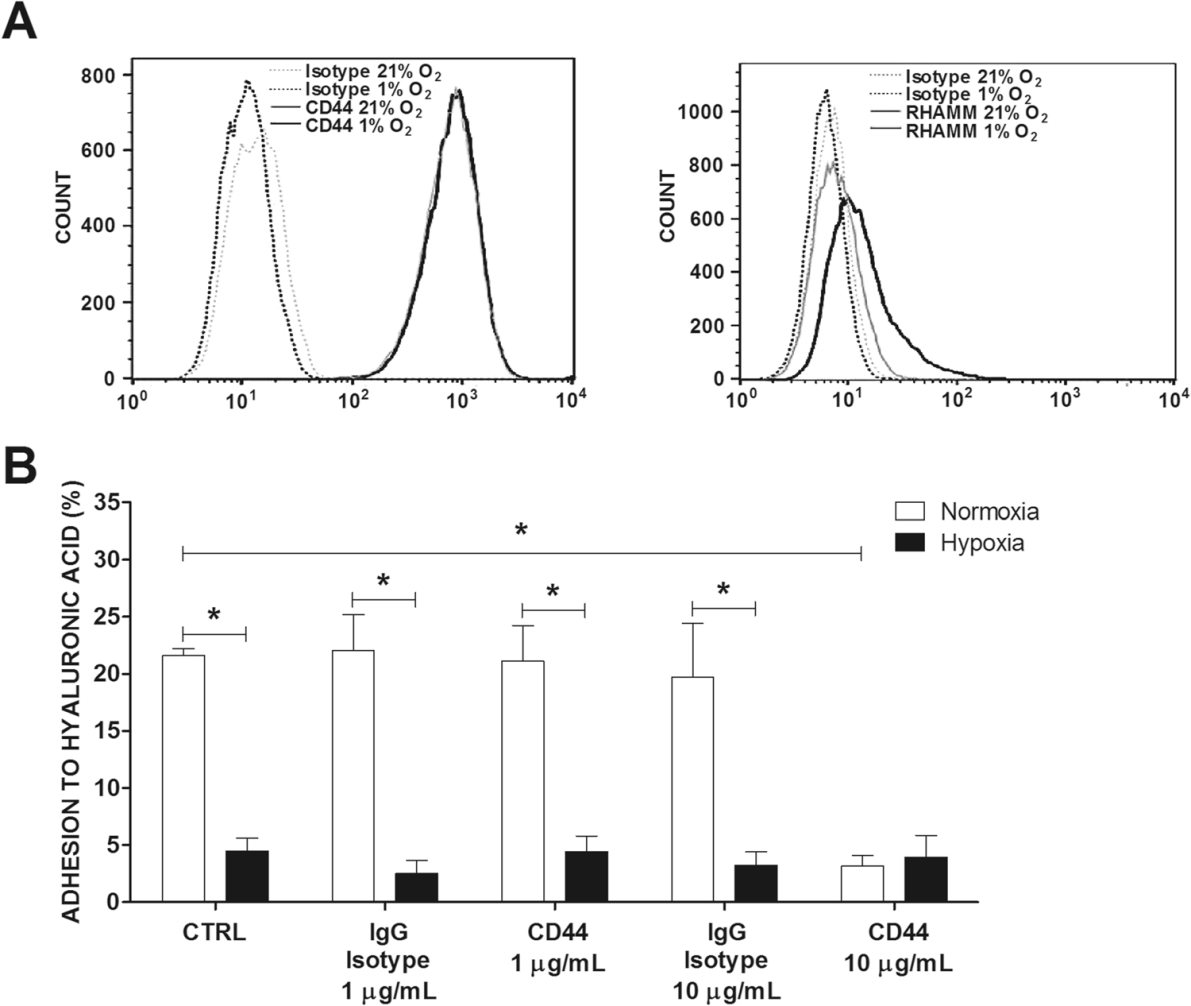
Surface expression of CD44 and RHAMM receptors in LAD2 mast cells and adhesion to hyaluronic acid (HA) after blocking CD44. Expression of CD44 and RHAMM on LAD2 cells was estimated after 1 h of incubation in 21% or 1% oxygen. A representative result was selected from six experiments (**A**). One-hour adhesion of LAD2 cells to HA in 21% (normoxia) and 1% (hypoxia) conditions after 30 min of preincubation with 1 or 10 μg/mL anti-CD44 antibody. CTRL, control (untreated cells) (**B**). Mean ± SEM. *P < 0.01, Student’s *t*-test with Bonferroni correction

First, we studied the surface expression of CD44 and HMMR/RHAMM in LAD2 MCs cultured under standard conditions and following short exposure to a hypoxic environment. As presented in Fig. 3A, there was high expression of CD44 on the surface of MCs that was not affected by 1-h incubation of MCs under hypoxic conditions. In contrast, RHAMM surface expression was almost imperceptible, suggesting that this HA receptor is not engaged in spontaneous adhesion of MCs to HA. Interestingly, MCs exposed to hypoxia presented an increased fluorescence signal following staining with anti-RHAMM Ab, suggesting induction of detectable surface expression of RHAMM under hypoxic conditions. Next, we tested the functional aspect of the observed high surface expression of CD44 in MCs by investigating the effect of blocking anti-CD44 Ab on MC adhesion to HA. As shown in Fig. 3B, anti-CD44 inhibited spontaneous MC adhesion to HA under standard conditions by 80%. Some residual MC adhesion to HA comparable to adhesion observed under hypoxic conditions was observed, even with a high excess of blocking anti-CD44 Ab. In a further attempt to investigate the potential mechanisms of the downregulation of MC adhesion to HA, we tested the effect of the hyaluronidase inhibitor hyaluromycin. Hyaluromycin at concentrations up to 2.5 µM did not affect MC adhesion to HA under normoxic or hypoxic conditions (Supplementary Table 1). Taken together, these data indicate that CD44 is at least in great part responsible for MC adhesion to HA under standard conditions and that inhibition of MC adhesion mediated by hypoxia is unlikely to result from changes in CD44 surface expression or HA degradation.

## Discussion

In this study, we observed spontaneous adhesion of LAD2 MCs to HA (Fig. 1A–C). This is similar to a report by Fukui et al. in which human MCs differentiated in vitro from CD34^+^ cells adhered to HA [12] and differs from observations that another human MC line, HMC-1, does not recognize HA as an adhesive substrate and adheres to this glycosaminoglycan to a very low extent [12, 30, 31]. The differences between LAD2 and HMC-1 cells in their adhesion to HA may reflect differences in their phenotypic characteristics, where LAD2 presents a more differentiated phenotype resembling primary human mast cell cultures. While T lymphocytes adhere to HA only upon activation [32, 33], MCs spontaneously adhere (Fig. 1A–C). Interestingly, we observed that such spontaneous MC adhesion was quickly downregulated by a decrease in oxygen concentration (Fig. 1A–C). Human MCs have been shown to adhere to several protein components of the ECM, including vitronectin [30, 34], laminin [30], and fibronectin [8, 30, 34]. In the case of these adhesion substrates, MC adhesion was upregulated by different stimuli, including cytokines and antigens. Recently, we have shown that hypoxia is another stimulus resulting in increased human MC adhesion to fibronectin [8]. In contrast to this observation, the current study demonstrated that hypoxia significantly inhibited MC adhesion to HA (Fig. 1A–C). This suggests that hypoxia is a significant factor modulating the interaction of MCs with the ECM, upregulating their adhesion to one component while downregulating adhesion to another. The effect of hypoxia on cell adhesion has been reported for other cell types and seems to be cell type- and adhesion substrate-specific, as hypoxia increases the adhesion of lymphocytes to mesenchymal cells [35] and neutrophils to the endothelium [36, 37] and inhibits the adhesion of several tumor cell lines to fibronectin, collagen type I, and vitronectin [38].

The effect of hypoxia on MC adhesion to HA was observed within minutes of decreasing oxygen concentration (Fig. 1C). Interestingly, even prolonged exposure of MCs to hypoxic conditions did not affect their adhesion to HA upon reoxygenation (Fig. 2A, B). Thus, a sufficiently high oxygen concentration seems to be necessary for MCs to adhere to HA, and a decrease in oxygen availability negatively regulates this adhesion process. Subsequent transfer of MCs from normoxic to hypoxic and back to normoxic conditions could also be perceived as an in vitro model relevant to the pathological process known as the ischemia–reperfusion injury that leads to damage to various organs and tissues [39]. There are reports of enhanced adhesion of immune cells following hypoxia and reoxygenation, such as lymphocytes adhering to fibroblasts [40] and neutrophils adhering to the endothelium [41]. In the case of MC adhesion to HA following hypoxia and reoxygenation, there was no enhancement of adhesion, and results were comparable to those observed under standard conditions.

Adhesion of LAD2 MCs to HA is mediated by CD44, as evidenced by its high expression (Table 2, Fig. 3A) and almost complete inhibition of the adhesion process by anti-CD44 Ab (Fig. 3B). This is aligned with the relatively high expression and major functional role of CD44 in interaction with HA previously observed in other MC types [12, 42] and is consistent with CD44 being a well-known receptor for this adhesion ligand engaged in adhesion of other immune cells to HA. The potential role of several alternative HA receptors in MC adhesion to HA is unlikely due to negligible (*LYVE-1*, *TLR2*, *TLR4*) or low (*ICAM-1*) genetic expression (Table 2). The exception was *RHAMM*, which exhibited substantial genetic expression- and surface expression-dependent oxygen concentration with hardly detectable presence on cell surface expression under standard conditions that was significantly upregulated following exposure of MCs to hypoxia (Fig. 3A). This oxygen-regulated surface expression of RHAMM is not consistent with the hypothesis of a direct role of RHAMM in mediating MC adhesion to HA, as this receptor seems to be virtually absent under conditions supporting strong adhesion of MCs to HA. The mechanism of downregulation of MC adhesion to HA under hypoxic conditions does not depend on the decreased number of CD44 on the MC surface, as surface expression did not differ between standard and hypoxic conditions (Fig. 3A). It is also unlikely that such downregulation is mediated by the degradation of HA. Hyaluronidase hydrolases that are responsible for the degradation of HA to its LMW form [29] were upregulated by hypoxia in cancer cells [43], and LAD2 MCs express a member of the hyaluronidase hydrolase family HYAL2 (Table 2). However, the presence of a hyaluronidase inhibitor did not prevent the downregulation of MC adhesion to HA by hypoxia (Supplementary Table 1). This negative observation, together with the lack of observable changes in the surface expression of HA receptors, suggests that hypoxia downregulates MC adhesion to HA by affecting HA receptor avidity. This is consistent with the model of conformational changes of the CD44 molecule from a low-affinity to a high-affinity state, leading to direct contact between HA and an arginine side chain in the polypeptide chain of CD44 [44]. Another potential element of the mechanism of downregulation of CD44-mediated MC adhesion to HA might involve RHAMM, which appeared on the MC surface exclusively under hypoxic conditions (Fig. 3A) and is known to colocalize with CD44 in the presence of HA [45]. It is worth noting that CD44 and RHAMM seem to have different roles in mediating the interaction of cells with HA, where CD44 preferentially mediates adhesion to HA and RHAMM preferentially mediates motility of this ECM component [46, 47]. Therefore, it is tempting to speculate that the observed change in the composition of these two major HA receptors on the MC surface under hypoxic conditions might result in a change in MC predisposition to strongly adhere and retain or migrate in sites of high abundancy of HA in the ECM under hypoxic conditions. Prolonged local hypoxia is a characteristic feature and important pathogenic factor of chronic inflammatory conditions [2–4]. Therefore, the effect of hypoxic conditions on mast cell adhesion to extracellular matrix might be a clinically relevant mechanism involved in local accumulation of mast cells observed, for example, in synovium in joints affected by rheumatoid arthritis [48] and in the stroma of solid tumor [49].

## Conclusion

In summary, we report here the observation of a rapid and drastic decrease in MC adhesion to HA following hypoxia as a result of an unknown mechanism that is likely dependent on conformational changes of the CD44 receptor. HA is a common component of the ECM that is engaged in inflammation, cancer, and wound healing, processes in which MCs play prominent roles. Regulation of MC adhesion to HA mediated by oxygen levels may provide a mechanism determining the infiltration and retainment of MCs at the site of inflammation, cancer, and tissue regeneration.

## Supplementary Information

Below is the link to the electronic supplementary material.

Supplementary file1 (DOC 40 KB)

## References

[1] Carreau A, El Hafny-Rahbi B, Matejuk A, Grillon C, Kieda C (2011). Why is the partial oxygen pressure of human tissues a crucial parameter? Small molecules and hypoxia. J Cell Mol Med.

[2] Eltzschig HK, Carmeliet P (2011). Hypoxia and inflammation. N Engl J Med.

[3] Finger EC, Giaccia AJ (2010). Hypoxia, inflammation, and the tumor microenvironment in metastatic disease. Cancer Metastasis Rev.

[4] Wilson WR, Hay MP (2011). Targeting hypoxia in cancer therapy. Nat Rev Cancer.

[5] Arriagada C, Silva P, Torres VA (2019). Role of glycosylation in hypoxia-driven cell migration and invasion. Cell Adh Migr.

[6] Gilkes DM, Bajpai S, Chaturvedi P, Wirtz D, Semenza GL (2013). Hypoxia-inducible factor 1 (HIF-1) promotes extracellular matrix remodeling under hypoxic conditions by inducing P4HA1, P4HA2, and PLOD2 expression in fibroblasts. J Biol Chem.

[7] Higgins DF, Kimura K, Iwano M, Haase VH (2008). Hypoxia-inducible factor signaling in the development of tissue fibrosis. Cell Cycle.

[8] Pastwińska J, Walczak-Drzewiecka A, Łukasiak M, Ratajewski M, Dastych J (2020). Hypoxia regulates human mast cell adhesion to fibronectin via the PI3K/AKT signaling pathway. Cell Adh Migr.

[9] Pastwińska J, Agier J, Dastych J, Brzezińska-Błaszczyk E (2017). Mast cells as the strength of the inflammatory process. Pol J Pathol.

[10] Theoharides TC, Alysandratos KD, Angelidou A, Delivanis DA, Sismanopoulos N, Zhang B, Asadi S, Vasiadi M, Weng Z, Miniati A, Kalogeromitros D (2012). Mast cells and inflammation. Biochim Biophys Acta.

[11] Misiak-Tłoczek A, Brzezińska-Błaszczyk E. Regulacja migracji komórek tucznych. Część 1: cząsteczki adhezji międzykomórkowej. Postepy Hig Med Dosw. 2007;61:485–492. 17909516

[12] Fukui M, Whittlesey K, Metcalfe DD, Dastych J (2000). Human mast cells express the hyaluronic-acid-binding isoform of CD44 and adhere to hyaluronic acid. Clin Immunol.

[13] Misra S, Heldin P, Hascall VC, Karamanos NK, Skandalis SS, Markwald RR, Ghatak S (2011). Hyaluronan-CD44 interactions as potential targets for cancer therapy. FEBS J.

[14] Yang M, Liu Y, Ren G, Shao Q, Gao W, Sun J, Wang H, Ji C, Li X, Zhang Y, Qu X (2015). Increased expression of surface CD44 in hypoxia-DCs skews helper T cells toward a Th2 polarization. Sci Rep.

[15] Liang G, Li S, Du W, Ke Q, Cai J, Yang J (2017). Hypoxia regulates CD44 expression via hypoxia-inducible factor-1α in human gastric cancer cells. Oncol Lett.

[16] Kirshenbaum AS, Akin C, Wu Y, Rottem M, Goff JP, Beaven MA, Rao VK, Metcalfe DD (2003). Characterization of novel stem cell factor responsive human mast cell lines LAD 1 and 2 established from a patient with mast cell sarcoma/leukemia; activation following aggregation of FcεRI or FcγRI. Leuk Res.

[17] Vandesompele J, De Preter K, Pattyn F, Poppe B, Van Roy N, De Paepe A, Speleman F (2002). Accurate normalization of real-time quantitative RT-PCR data by geometric averaging of multiple internal control genes. Genome Biol..

[18] Wang SJ, Wong G, de Heer AM, Xia W, Bourguignon LY (2009). CD44 variant isoforms in head and neck squamous cell carcinoma progression. Laryngoscope.

[19] He X, Liao W, Li Y, Wang Y, Chen Q, Jin J, He S (2015). Upregulation of hyaluronan-mediated motility receptor in hepatocellular carcinoma predicts poor survival. Oncol Lett.

[20] Kurihara Y, Furue M (2013). Interferon-γ enhances phorbol myristate acetate-induced cell attachment and tumor necrosis factor production via the NF-κB pathway in THP-1 human monocytic cells. Mol Med Rep.

[21] Lu Y, Yang Q, Du Y, Feng G, Yang C (2007). Expression analysis of lymphangiogenic factors in human colorectal cancer with quantitative RT-PCR. Cancer Invest.

[22] Hopkins PA, Fraser JD, Pridmore AC, Russell HH, Read RC, Sriskandan S (2005). Superantigen recognition by HLA class II on monocytes up-regulates toll-like receptor 4 and enhances proinflammatory responses to endotoxin. Blood.

[23] Wu Z, Zhang L (2017). Polycomb group proteins: novel molecules associated with ultraviolet A-induced photoaging of human skin. Exp Ther Med.

[24] Nanashima N, Horie K, Maeda H, Tomisawa T, Kitajima M, Nakamura T (2018). Blackcurrant anthocyanins increase the levels of collagen, elastin, and hyaluronic acid in human skin fibroblasts and ovariectomized rats. Nutrients.

[25] Untergasser A, Cutcutache I, Koressaar T, Ye J, Faircloth BC, Remm M, Rozen SG (2012). Primer3–new capabilities and interfaces. Nucleic Acids Res.

[26] Ye J, Coulouris G, Zaretskaya I, Cutcutache I, Rozen S, Madden TL (2012). Primer-BLAST: a tool to design target-specific primers for polymerase chain reaction. BMC Bioinformatics.

[27] Litwiniuk M, Krejner A, Speyrer MS, Gauto AR, Grzela T (2016). Hyaluronic acid in inflammation and tissue regeneration. Wounds.

[28] Weigel JA, Raymond RC, Weigel PH (2002). The hyaluronan receptor for endocytosis (HARE) is not CD44 or CD54 (ICAM-1). Biochem Biophys Res Commun.

[29] Asada M, Sugie M, Inoue M, Nakagomi K, Hongo S, Murata K, Irie S, Takeuchi T, Tomizuka N, Oka S (1997). Inhibitory effect of alginic acids on hyaluronidase and on histamine release from mast cells. Biosci Biotechnol Biochem.

[30] Krüger-Krasagakes S, Grützkau A, Baghramian R, Henz BM (1996). Interactions of immature human mast cells with extracellular matrix: expression of specific adhesion receptors and their role in cell binding to matrix proteins. J Invest Dermatol.

[31] Trautmann A, Feuerstein B, Ernst N, Bröcker EB, Klein C (1997). Heterotypic cell-cell adhesion of human mast cells to fibroblasts. Arch Dermatol Res.

[32] Dillon PW, Keefer K, Blackburn JH, Houghton PE, Krummel TM (1994). The extracellular matrix of the fetal wound: hyaluronic acid controls lymphocyte adhesion. J Surg Res.

[33] Galluzzo E, Albi N, Fiorucci S, Merigiola C, Ruggeri L, Tosti A, Grossi CE, Velardi A (1995). Involvement of CD44 variant isoforms in hyaluronate adhesion by human activated T cells. Eur J Immunol.

[34] Kulka M, Metcalfe DD (2006). TLR3 activation inhibits human mast cell attachment to fibronectin and vitronectin. Mol Immunol.

[35] Ginis I, Mentzer SJ, Faller DV (1993). Hypoxia induces lymphocyte adhesion to human mesenchymal cells via an LFA-1-dependent mechanism. Am J Physiol..

[36] Lynch EM, Moreland RB, Ginis I, Perrine SP, Faller DV (2001). Hypoxia-activated ligand HAL-1/13 is lupus autoantigen Ku80 and mediates lymphoid cell adhesion in vitro. Am J Physiol Cell Physiol.

[37] Wood JG, Mattioli LF, Gonzalez NC (1999). Hypoxia causes leukocyte adherence to mesenteric venules in nonacclimatized, but not in acclimatized, rats. J Appl Physiol (1985).

[38] Hasan NM, Adams GE, Joiner MC, Marshall JF, Hart IR (1998). Hypoxia facilitates tumour cell detachment by reducing expression of surface adhesion molecules and adhesion to extracellular matrices without loss of cell viability. Br J Cancer.

[39] Seibert AF, Haynes J, Taylor A (1993). Ischemia-reperfusion injury in the isolated rat lung. Role of flow and endogenous leukocytes. Am Rev Respir Dis..

[40] Han MK, Kim JS, Park BH, Kim JR, Hwang BY, Lee HY, Song EK, Yoo WH (2003). NF-kappaB-dependent lymphocyte hyperadhesiveness to synovial fibroblasts by hypoxia and reoxygenation: potential role in rheumatoid arthritis. J Leukoc Biol.

[41] Palluy O, Morliere L, Gris JC, Bonne C, Modat G (1992). Hypoxia/reoxygenation stimulates endothelium to promote neutrophil adhesion. Free Radic Biol Med.

[42] Takano H, Nakazawa S, Shirata N, Tamba S, Furuta K, Tsuchiya S, Morimoto K, Itano N, Irie A, Ichikawa A, Kimata K, Nakayama K, Sugimoto Y, Tanaka S (2009). Involvement of CD44 in mast cell proliferation during terminal differentiation. Lab Invest.

[43] Gao F, Okunieff P, Han Z, Ding I, Wang L, Liu W, Zhang J, Yang S, Chen J, Underhill CB, Kim S, Zhang L (2005). Hypoxia-induced alterations in hyaluronan and hyaluronidase. Adv Exp Med Biol.

[44] Jamison FW, Foster TJ, Barker JA, Hills RD, Guvench O (2011). Mechanism of binding site conformational switching in the CD44-hyaluronan protein-carbohydrate binding interaction. J Mol Biol.

[45] Carvalho AM, Soares da Costa D, Paulo PMR, Reis RL, Pashkuleva I (2021). Co-localization and crosstalk between CD44 and RHAMM depend on hyaluronan presentation. Acta Biomater.

[46] Masellis-Smith A, Belch AR, Mant MJ, Turley EA, Pilarski LM (1996). Hyaluronan-dependent motility of B cells and leukemic plasma cells in blood, but not of bone marrow plasma cells, in multiple myeloma: alternate use of receptor for hyaluronan-mediated motility (RHAMM) and CD44. Blood.

[47] Leng Y, Abdullah A, Wendt MK, Calve S (2019). Hyaluronic acid, CD44 and RHAMM regulate myoblast behavior during embryogenesis. Matrix Biol.

[48] Pitzalis C, Kelly S, Humby F (2013). New learnings on the pathophysiology of RA from synovial biopsies. Curr Opin Rheumatol.

[49] Cimpean AM, Tamma R, Ruggieri S, Nico B, Toma A, Ribatti D (2017). Mast cells in breast cancer angiogenesis. Crit Rev Oncol Hematol.

