# Supplementary material for: Hypoxia modulates human mast cell adhesion to hyaluronic acid
Source: Immunol Res. 2021 Nov 17;70(2):152–60. doi: 10.1007/s12026-021-09228-x (PMC8917009; doi:10.1007/s12026-021-09228-x)
Supplement: Supplementary file 1 — Supplementary file1 (DOC 40 KB) [file 12026_2021_9228_MOESM1_ESM.doc]

**Table S1. Adhesion of LAD2 to HA after blocking hyaluronidases. Percentage of adhesion inhibition after 20 hrs of preincubation with 2.5 μM of Hyaluromycin (HyalMyc) in 21% (Normoxia) and 1% (Hypoxia) oxygen relative to the carrier (0.1% DMSO).**

|  | Inhibition of adhesion (%) | |
| --- | --- | --- |
|  | Normoxia | Hypoxia |
| DMSO | 0 | 0 |
| HyalMyc | -4.63 | -4.95 |
